# Genome-wide association study of varicose veins identifies a protective missense variant in *GJD3* enriched in the Finnish population

**DOI:** 10.1038/s42003-022-04285-w

**Published:** 2023-01-18

**Authors:** Pyry Helkkula, Shabbeer Hassan, Elmo Saarentaus, Emilia Vartiainen, Sanni Ruotsalainen, Jaakko T. Leinonen, FinnGen, Aarno Palotie, Juha Karjalainen, Mitja Kurki, Samuli Ripatti, Taru Tukiainen

**Affiliations:** 1 Institute for Molecular Medicine Finland (FIMM), University of Helsinki, Helsinki, Finland; 2 Program in Medical and Population Genetics and Stanley Center for Psychiatric Research, Broad Institute of Harvard and MIT, Cambridge, MA USA; 3 Analytic and Translational Genetics Unit, Massachusetts General Hospital, Boston, MA USA; 4 Broad Institute of the Massachusetts Institute of Technology and Harvard University, Cambridge, MA USA; 5 Department of Public Health, Clinicum, Faculty of Medicine, University of Helsinki, Helsinki, Finland

**Keywords:** Genome-wide association studies, Target identification, Genetics research

## Abstract

Varicose veins is the most common manifestation of chronic venous disease that displays female-biased incidence. To identify protein-inactivating variants that could guide identification of drug target genes for varicose veins and genetic evidence for the disease prevalence difference between the sexes, we conducted a genome-wide association study of varicose veins in Finns using the FinnGen dataset with 17,027 cases and 190,028 controls. We identified 50 associated genetic loci (*P* < 5.0 × 10^−8^) of which 29 were novel including one near *ERG* with female-specificity (rs2836405-G, OR[95% CI] = 1.09[1.05–1.13]*, P* = 3.1 × 10^−8^). These also include two X-chromosomal (*ARHGAP6* and *SRPX*) and two autosomal novel loci (*TGFB2* and *GJD3*) with protein-coding lead variants enriched above 56-fold in Finns over non-Finnish non-Estonian Europeans. A low-frequency missense variant in *GJD3* (p.Pro59Thr) is exclusively associated with a lower risk for varicose veins (OR = 0.62 [0.55–0.70], *P* = 1.0 × 10^−14^) in a phenome-wide scan of the FinnGen data. The absence of observed pleiotropy and its membership of the connexin gene family underlines *GJD3* as a potential connexin-modulating therapeutic strategy for varicose veins. Our results provide insights into varicose veins etiopathology and highlight the power of isolated populations, including Finns, to discover genetic variants that inform therapeutic development.

## Abstract (web summary)

A genome-wide association study in a Finnish cohort identifies several new loci associated with varicose veins, including the connexin gene family member, *GJD3*, as a future druggable target.

## Introduction

Varicose veins (VV) is one of the most common clinical manifestations of chronic venous disease that afflicts approximately 30% of the population in Western countries^1,2^. For reasons largely unknown, VV appears to display a female bias^3–8^, yet conflicting evidence regarding the VV sex difference also exists^2,9–12^. VV predisposes to serious complications, such as deep vein thrombosis (DVT) and pulmonary embolism^13,14^. Despite these ramifications, the treatment options for VV remain limited.

Heritability is estimated to account for approximately 18% of the individual variation in VV risk^15–18^, and accordingly genome-wide association studies (GWAS) have identified over 50 genetic loci associated with VV^16–20^. For most of the identified VV-loci the putative causal gene nevertheless remains unknown hence complicating the translation of the genetic findings into therapeutic strategies. However, for several complex traits the study of low-frequency protein-coding variation potentially enriched in population isolates such as Finns has yielded novel loci pointing directly to a likely causal gene and identifying possible intervention opportunities^21,22^.

Population isolates, such as Finns, show an enrichment of protein-coding variants compared to admixed populations^23–28^. Genetic studies of population isolates thus boost the statistical power to detect associations between phenotypes and protein-coding variants and have identified novel associations between variants and phenotypes^21,22,24–26,28,29^. Studies of genotyped individuals coupled with comprehensive healthcare records have the potential to identify disease etiology and likely causal relationships between traits. Phenome-wide association studies (PheWASs) can be used to study the pleiotropy of likely clinically relevant variants uncovered in a GWAS and can generate new hypotheses that provide insight into the genetic architecture of complex traits.

Given these findings, we asked the following questions: first, could we leverage the unique Finnish genetics to find novel loci with protein-coding variants associated with VV, second, could we provide new biological insights through these novel loci and genes to propose new potential VV drug target genes, and third, could we explain the sex-based differences in VV incidence in part using genetic factors?

To address the three questions posed above, we used the FinnGen cohort, consisting of 218,792 genotyped individuals, of which 17,027 were VV cases identified through linkage to the nationwide healthcare records to test which genetic variants were associated with VV. We carried out GWASs together and separately for the sexes to identify sex-specific associations. To gain insight into the etiology of VV, we first conducted PheWASs in the identified VV-associated loci using 1706 clinical endpoints. Subsequently, we computed the genetic correlations between the significantly associated disease endpoints in the PheWASs. To try to identify the causal genetic variants among the VV associated loci, we carried out both genetic finemapping and VV pathology-relevant tissue colocalization analyses of these loci. Finally, we investigated what disease-relevant etiologies the shortlisted genes could have.

## Results

### FinnGen Study cohort

We used the FinnGen Study data (release 5) as our discovery cohort for finding genetic variants associated with VV. The FinnGen data included 218,792 genotyped Finns and their healthcare data from nationwide healthcare registries with a follow-up from 1969 until the end of 2018. Using the hospital discharge registry and cause of death registry we identified 17,027 VV cases (13,045 females and 3982 males) in the FinnGen data (see Methods for exact ICD-based case definition). The mean age of the first diagnosis was 45.0 years (Table 1). As controls in our VV association analyses we used 190,028 vein and lymphatic disease free FinnGen participants (see “Methods” for further details).

**Table 1:** The VV study cohort summary of the fifth data release of FinnGen.

	All	Females	Males
No. of individuals^a^	207,055	117,032	90,023
No. of cases	17,027	13,045	3982
Unadjusted VV prevalence (%)	7.9	10.6	4.2
Mean-age at first VV disease event (years old)	45.0	43.6	49.6

^a^The total number of VV cases and vein- and lymphatic disease-free controls.

### Association of genetic variants with VV

**Fig. 1:**
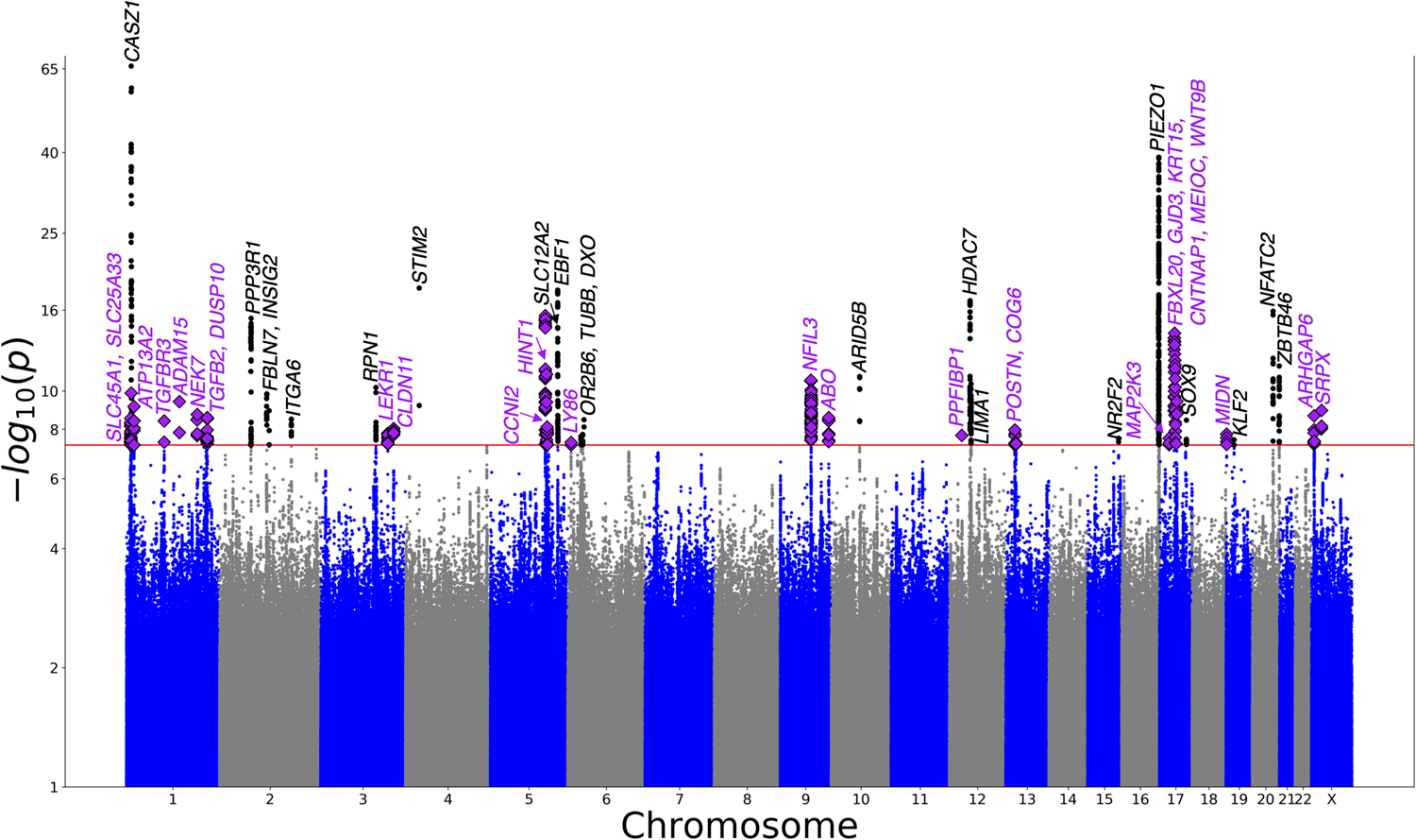
VV Manhattan plot. Summary of the VV GWAS results from the sex-combined GWAS of the FinnGen cohort showing the −log_10_-transformed *P* value of each tested variant along the logarithmic vertical axis and base-pair positions along the chromosomes along the horizontal axis. The genome-wide significant variants in novel and known loci are indicated by diamonds in magenta and black points respectively. The red line signifies the genome-wide significance threshold of *P* = 5.0 × 10^−8^.

We conducted a genome-wide association study of VV using the FinnGen data including 13,112,471 autosomal and 428,995 X-chromosomal variants with minor allele frequency (MAF) > 0.001. We identified genome-wide significantly associated variants (*P* < 5.0 × 10^−8^) at 50 genetic loci (Fig. 1 and Supplementary Data 1). These included 21 previously reported loci, our analysis replicating, for instance, associations for VV near *PIEZO1, SOX9, ADAM15* and *CASZ1*^16–20^. We identified 29 novel VV loci (Table 2), which includes two X-chromosomal loci for VV (*ARHGAP6* and *SPRX*), and *TGFB2* and *TGFBR3* which belong to the transforming growth factor beta (TGF-β) signaling pathway. In two of our novel loci (*TGFB2* and *GJD3*) the lead SNP was over 56-fold enriched in Finns compared to non-Finnish non-Estonian Europeans (NFEEs) in the gnomAD genome reference database^30^ (Table 2) and in five novel loci (*TGFB2*, *MAP2K3*, *GJD3, CNTNAP1*, and *SRPX*) the lead variant is protein-coding.

**Table 2:** Summary of the VV lead SNPs for novel loci and their colocalized genes.

Lead variant	Chr:pos:ref/alt^a^	Alt AF^b^	Effect (*β*)^c^	*P*	Variant type^d^	Finnish enrichment^e^	Nearest genes	Colocalized gene expression^f^
rs17032325	1:8238488:G/A	0.095	0.128	1.6 × 10^−8^	Regulatory region	3.41	*SLC45A1*	–
rs76077063	1:9471381C/T	0.046	0.189	2.9 × 10^−9^	Intergenic	15.68	*SLC25A33*	*SPSB1*
rs3738814	1:17005181:A/G	0.484	−0.083	7.2 × 10^−10^	Intron	1.19	*ATP13A2*	*MFAP2*
rs6662887	1:91709357:C/T	0.297	−0.087	4.0 × 10^−9^	Intron	0.92	*TGFBR3*	–
rs11589479	1:155060832:G/A	0.139	0.121	4.0 × 10^−10^	Splice region	0.89	*ADAM15*	–
rs72749413	1:198091159:C/T	0.129	−0.12	1.9 × 10^−9^	Intergenic	1.33	*NEK7*	*NEK7*
rs192335285	1:218434159:C/T	2.54e-3	0.77	2.5 × 10^−8^	Synonymous	56.13	*TGFB2*	–
rs11118774	1:221612617:G/A	0.398	−0.081	2.7 × 10^−9^	Regulatory	1.31	*DUSP10*	–
rs10049210	3:157079584.T/C	0.332	−0.081	1.5 × 10^−8^	Upstream gene	0.80	*LEKR1*	*TIPARP*
rs493410	3:170754426:G/T	0.252	0.089	9.0 × 10^−9^	Intron	1.34	*CLDN11*	–
rs2189759	5:131047353:G/A	0.150	0.13	9.4 × 10^−12^	Intergenic	2.41	*HINT1*	–
rs708461	5:132751125:G/C	0.201	0.10	7.7 × 10^−9^	3-prime UTR	2.49	*CCNI2, SEPT8*	*AFF4, HINT1, KIF3A*
rs1294414	6:6740133:A/G	0.641	−0.077	3.9 × 10^−8^	Intergenic	1.01	*LY86*	–
rs1981030	9:91455019:T/C	0.244	0.10	2.2 × 10^−11^	Intergenic	1.50	*NFIL3*	–
rs635634	9:133279427:T/C	0.801	−0.010	2.7 × 10^−9^	Upstream gene	0.99	*ABO*	–
rs7295096	12:27644972:A/G	0.827	0.10	1.9 × 10^−8^	Intron	1.06	*PPFIBP1*	–
rs7321631	13:37543414:T/C	0.552	−0.08	1.1 × 10^−8^	Intron	1.12	*POSTN*	*POSTN*
rs4403927	13:39767008:G/T	0.362	−0.079	4.0 × 10^−8^	Intron	0.91	*COG6*	–
rs1657686	17:21313652:T/C	0.502	−0.0749	2.6 × 10^−8^	Missense	1.21	*MAP2K3*	–
rs76327840	17:39247659:G/C	0.0137	−0.44	1.8 × 10^−13^	Intron	28.0	*FBXL20*	–
rs201955556	17:40363641:G/T	0.014	−0.47	1.0 × 10^−14^	Missense	69.16	*GJD3*	–
rs200702391	17:41515873:G/C	0.0153	−0.44	2.3 × 10^−12^	Intron	51.9	*KRT16*	–
rs149387021	17:42696125:C/T	0.0156	−0.32	5.9 × 10^−9^	Synonymous	29.0	*CNTNAP1*	–
rs182510184	17:44645052:A/G	0.0272	−0.24	4.4 × 10^−8^	Upstream gene	49.1	*MEIOC*	–
rs2165846	17:46864000:A/G	0.482	0.084	3.8 × 10^−10^	Intron	1.20	*WNT9B*	–
rs3746106	19:1250110:C/A	0.424	−0.077	1.7 × 10^−8^	5-prime UTR	0.98	*MIDN*	–
rs2836405^g^	21:38444848:G/A	0.536	0.086	3.1 × 10^−8^	Intron	1.03	*ERG*	–
rs5979390	23:11277189:C/T	0.29	−0.079	2.2 × 10^−9^	Intron	1.34	*ARHGAP6*	*ARHGAP6*
rs35318931	23:38149868:G/A	0.062	−0.15	1.2 × 10^−9^	Missense	0.69	*SRPX*	–

Novel susceptibility loci genome-wide significantly associated (*P* < 5 × 10^−8^) with varicose veins are shown.

^a^Lead variant position is given in genome build GRCh38.

^b^The reported alternative allele frequency (AF) is observed in the FinnGen data.

^c^The associated effect (*β*) of each variant is the logarithm of the odds ratio.

^d^The variant types we obtained using the Ensembl Variant Effect Predictor^72^.

^e^The Finnish enrichment of each lead variant is with respect to non-Finnish non-Estonian Europeans (NFEEs) in the gnomAD database^30^, v2.1.1.

^f^Colocalized gene expression refers to the genes with a *cis*-eQTL variant in GTEx V8 tissues considered relevant to VV etiopathology (adipose, arterial and nerve tissues)^16,17^, such that within a locus the associations with gene expression levels and varicose veins risk were shared for variant with a high posterior probability (PP.H4 > 0.8).

^g^Female-specifically associated lead variant.

Multiple pieces of evidence that lend support to the validity of our novel GWAS findings. First, the observed-scale SNP-based heritability, estimated from the GWAS summary statistics using LD score regression (LDSC)^31,32^, was *h*^2^_obs_ = 5.82 [4.7–7.0]%, and in line with previous studies done in the UK Biobank^16–18^. Second, The genetic correlation based on LDSC was 90.0% (90.0 [80.7–98.5]%, *P* = 3.9 × 10^−87^) between FinnGen and UK Biobank VV association statistics (*N* = 401,656; both males and females) demonstrating good concordance. Third, the strong and highly significant correlation between the associated effects of 37 sex-shared VV lead variants in FinnGen common for UK Biobank and FinnGen data (*r* = 0.92, *P* = 3.6 × 10^−16^; Supplementary Fig. 1) implies a high concordance between the VV ascertainment for the two cohorts. Fourth, the sign-test for the 18 sex-shared novel FinnGen lead variants also found in UK Biobank was statistically significant (two-tailed *P* = 7.6 × 10^−6^, i.e., no lead variants with a discordant sign). Finally, 14 of the 18 novel lead variants replicated with a concordant effect direction and a P-value below 1.3 × 10^−3^ in the UK Biobank (two-tailed P-value with a Bonferroni-correction for 37 tests)^17^ (Supplementary Data 1).

We also finemapped the VV-associated loci to pinpoint potential causal variants and genes. Using SuSiE^33^ we identified 53 95% credible sets in 47 loci outside the HLA region (Supplementary Data 2). To assess the probability of causality among the protein-coding lead variants we inspected their posterior inclusion probabilities (PIPs). The novel loci with protein-coding lead variants also had high PIPs, except for the *CNTNAP1* lead variant; *TGFB2* (rs192335285-T): 0.489, *MAP2K3* (rs1657686-C): 0.273, *GJD3* (rs201955556-T): 0.450, *CNTNAP1* (rs149387021-T): 8.13 × 10^−7^ and *SRPX* (rs35318931-A): 0.687.

### Sex-specific analyses

**Fig. 2:**
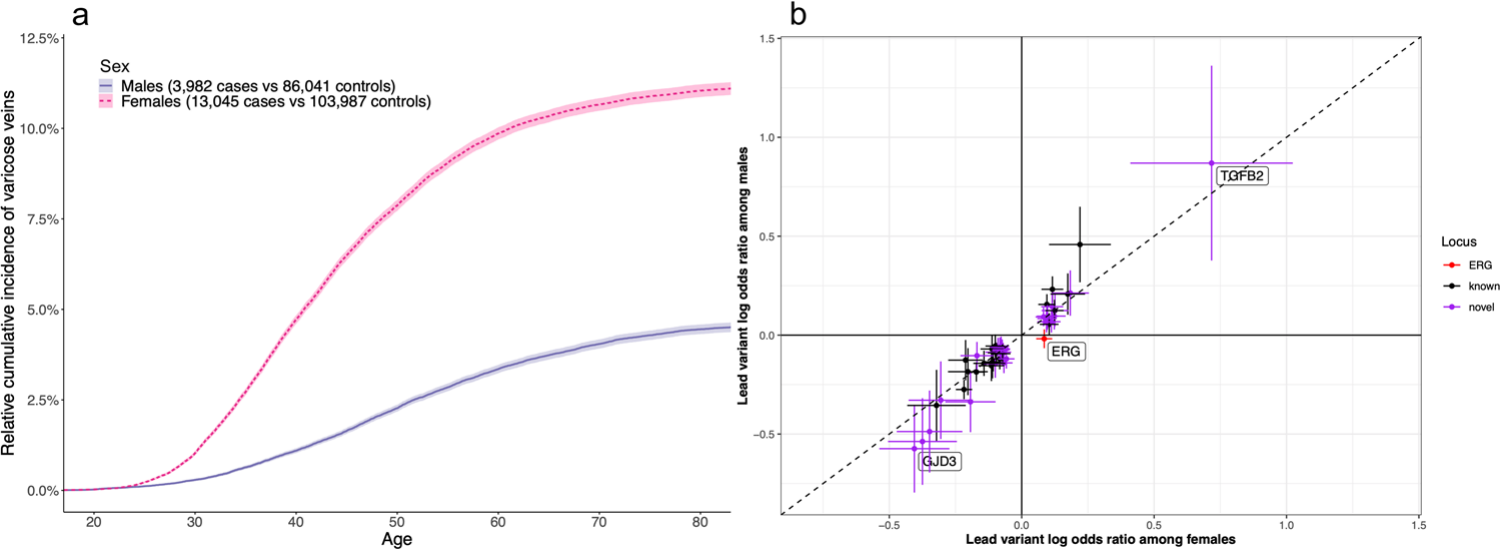
Sex-based differences in the incidence and lead variants of VV in FinnGen. **a** The point estimates of the relative VV incidence in the FinnGen cohort as a function of age years for each sex are shown. The shadings around the incidence curves denote the 95% confidence intervals of the point estimates. **b** A correlation plot of the identified VV lead SNPs’ logarithm of odds ratios and their standard errors between the sexes are given.

As with many epidemiological studies on varicose veins^2,3^, we found major differences between sexes (Table 1 and Fig. 2a) regarding prevalence (unadjusted percentage, females =10.6 [10.1–11.2]%, males = 4.2 [3.6–4.8]%, *Z* = 12.3, *P* < 1× 10^−4^), mean age at first event (females = 43.61 years, males = 49.55 years, *P* < 1 × 10^−4^). We performed VV GWASs separately for FinnGen males (3982 cases and 86,041 controls) and females (13,045 cases and 103,987 controls) to investigate whether genetic data could provide insights into the established sex difference in VV incidence. The sex-specific *h*^2^_obs_ in the FinnGen data was 6.8 [5.0–8.6]% for females and 4.4 [2.7–6.1]% for males. The difference in heritability was non-significant (*Z* = 1.95, *P* = 0.051) which suggests little sex-based difference in genetic variance for VV. The genetic correlation (*r*_g_) between females and males was indistinguishable from 100% (*r*_g_ = 100 [80–130]%, *P* = 2.6 × 10^−16^) pointing to largely shared genetic determinants for VV between the sexes. We additionally observed a high concordance in the sex-specific effects of the lead variants from the sex-combined analysis (Fig. 2b and Supplementary Data 3).

We nevertheless identified one new genome-wide significant locus in the female-specific GWAS that was not significant in the sex-combined analysis; rs2836405-G near *ERG* was associated with a higher risk for VV in females (OR = 1.09 [1.05–1.13], *P* = 3.1 × 10^−8^) but not in males (OR = 0.98 [0.93–1.03], *P* = 0.45) (difference tested under χ^2^ with degrees of freedom = 1, *P* = 3.3 × 10^−4^). The *ERG* locus reached genome-wide significance in an earlier sex-combined VV GWAS with UK Biobank^17^ (OR = 1.06 [1.04–1.08], *P* = 1.3 × 10^−9^) but failed the replication step in the 23andMe cohort and thus we consider the *ERG* locus in this study novel. Given the overall similarity of VV genetics in males and females, we used the results from the sex-combined GWAS in our subsequent analyses unless otherwise stated.

### VV lead variants with pleiotropy

**Fig. 3:**
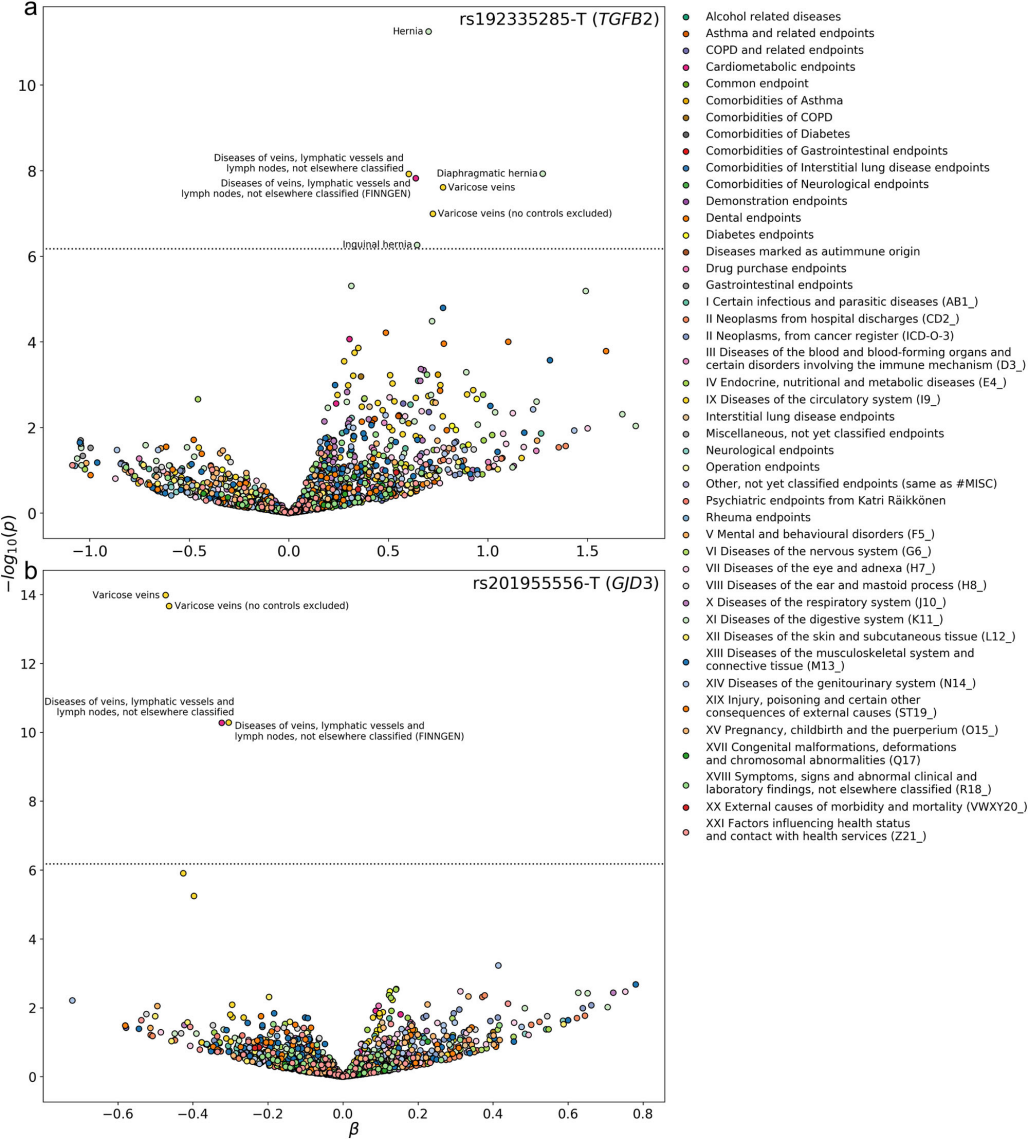
*TGFB2* and *GJD3* locus zoom plots. The plot shows the VV associations for all analyzed variants within a 250 kb window around the lead variants of *TGFB2* in **a** and *GJD3* in **b** loci with −log_10_-transformed *P* values along the vertical axis and the chromosome position along the horizontal axis. Genes overlapping each locus are also displayed. Variants are colored by their LD value (*r*^2^) ranges calculated from the SISu v3 genome reference panel^69^ in reference to the lead SNP in each region indicated by a purple diamond.

To test for the possible pleiotropy of the VV-associated loci, we performed phenome-wide association analyses (PheWASs) of the 48 lead SNPs not in the *HLA* region (chr6:28,510,120–33,480,577). The phenome-wide scans included 1706 disease endpoints with a minimum of 500 cases in the FinnGen data. In the sex-combined analysis eight lead SNPs of which six were novel displayed phenome-wide significant associations (two-sided *P* < 6.1 × 10^−7^; Bonferroni correction for 81,880 tests) with disease endpoints besides various disorders of veins (Supplementary Data 4). For these eight VV lead variants we replicated 23 out of 48 phenome-wide significant associations by testing for genetic colocalization with the same variant (posterior probability for colocalization with the same causal variant being above 0.8 [PP.H4 > 0.8)]). The lead SNPs in three novel loci displayed phenome-wide associations and colocalizations with various hernia endpoints including *LY86* (rs1294414-G; inguinal hernia; PP.H4 = 0.92; OR = 0.92 [0.89–0.94], *P* = 1.6 × 10^−12^) and *SRPX* (rs35318931-A; inguinal hernia; PP.H4 = 1.00; OR = 0.90 [0.87–0.94], *P* = 7.4 × 10^−8^). The *TGFB2* locus was also phenome-wide significantly associated with hernia endpoints (rs192335285-T; diaphragmatic hernias, OR = 3.58 [2.31–5.57], *P* = 1.2 × 10^−8^) (Fig. 3a and the *rs192335285–TGFB2* tab in Supplementary Data 4) but these associations were not fully supported by the colocalization analysis (rs192335285-T; diaphragmatic hernia, PP.H3 = 0.49, PP.H4 = 0.21). For the lead variant (rs635634-C; VV; OR = 0.91 [0.87–0.94], *P* = 2.7 × 10^−9^) in the particularly pleiotropic *ABO* locus we confirmed its pleiotropy with 13 out of 26 disorders, including cholelithiasis (OR = 0.91 [0.89–0.94], *P* = 1.0 × 10^−9^; PP.H4 = 0.99) and cardiovascular diseases (OR = 0.94 [0.92–0.96], *P* = 2.4 × 10^−10^; PP.H4 = 0.99). The lead SNP in the other known locus *ZBTB46* (rs150989585-T; VV; OR = 0.70 [0.63–0.78], *P* = 2.8 × 10^−12^) was associated with a higher risk for cerebral aneurysms (OR = 2.45 [1.73–3.45], *P* = 3.2 × 10^−7^; PP.H4 = 0.96). The novel *ERG* locus variant identified in the female-specific analysis was not phenome-wide significantly associated with any other diseases than VV.

### Genetic correlations of VV with other traits

We computed the cross-trait genetic correlations between VV and 31 other traits that were phenome-wide significantly associated with the lead variants of the VV GWAS loci (Supplementary Data 5). VV was statistically significantly correlated (two-tailed *P* < 1.6 × 10^−3^; Bonferroni correction with 31 traits) genetically, first, with venous thromboembolism (VTE) (*r*_g_ = 34.1 [16.1–52.2]%, *P* = 2 × 10^−4^], deep vein thrombosis of lower extremities (*r*_g_ = 38.5 [16.3–60.7]%, *P* = 7 × 10^−4^), phlebitis and thrombophlebitis (*r*_g_ = 58.3 [28.6–88.0]%, *P* = 1 × 10^−4^), findings which are concordant with Shadrina et al.^18^, and, second, with cardiovascular diseases (*r*_g_ = 39.9 [30.0–49.8]%, *P* = 3.3 × 10^−15^). The repeatedly shared loci between VV and inguinal hernias were, however, not observed as a genome-wide correlation (*r*_g_ = 5.73 [−6.03–17.5]%, *P* = 0.34).

### Gene-level colocalization analyses in relevant tissue types

To improve our understanding of the biological mechanisms of the discovered loci, we performed colocalization analyses with the 50 VV loci and gene expression in etiopathologically relevant tissue types (i.e., adipose, artery, and nerve)^16,17^ from the GTEx data set^34^ using *coloc*^35^. In 18 of the 50 VV loci we identified 26 unique colocalized genes in relevant tissue types (posterior probability for shared causal variant, PP.H4 > 0.8) (Supplementary Data 1 and 6). In the novel loci, *ARHGAP6* expression coincided with the *ARHGAP6* locus in the X chromosome (PP.H4 > 0.91 in adipose subcutaneous) and in the *ATP13A2* locus *MFAP2* expression quantitative trait loci (eQTLs) colocalized in adipose, arterial and nerve tissue with VV risk (Supplementary Data 1 and 6). Additionally, we observed that the VV-associated *NEK7* locus was colocalized with *NEK7* expression (PP.H4 = 0.86 in aortic artery) and that *POSTN* eQTLs (PP.H4 = 0.86 in aortic artery) was colocalized with the *POSTN* locus. In seven of the previously reported loci (Supplementary Data 1 and 6), the colocalization analyses showed a single candidate gene. Examples of this include *ITGA6* (PP.H4 = 0.87 in tibial nerve), *INSIG2* (colocalization in three different tissues), and *FBLN7* (colocalization in six different tissues). Overall, in the 13 loci with a single colocalizing gene, in 8 instances the results pointed to the gene residing closest to the lead variant of the locus.

### Finnish-enriched VV-associated lead variants

Exemplifying the power of isolated populations for discovering new genetic associations with complex diseases like VV, we observed that the lead variants in 22 novel loci were enriched in Finns compared to NFEEs (Supplementary Data 1 and Table 2). In two of the novel loci the lead SNPs were more than 56-fold enriched in the FinnGen data compared to the NFEEs (Table 2); a rare synonymous variant in *TGFB2* (rs192335285-T, MAF in Finns = 2.54 × 10^−3^, Finnish Enrichment = 56.13) and a low-frequency missense variant in *GJD3* (rs201955556-T, MAF in Finns = 0.0135, Finnish Enrichment = 69.16). We scrutinized these two protein-coding variants for mechanistic insights. Furthermore, both the variants had large associated effect sizes (Table 2, Fig. 2b, and Supplementary Data 1) that substantiate possible efficacious therapeutic targeting of these genes against VV.

### *TGFB2*, TGF-β signaling and VV

**Fig. 4:**
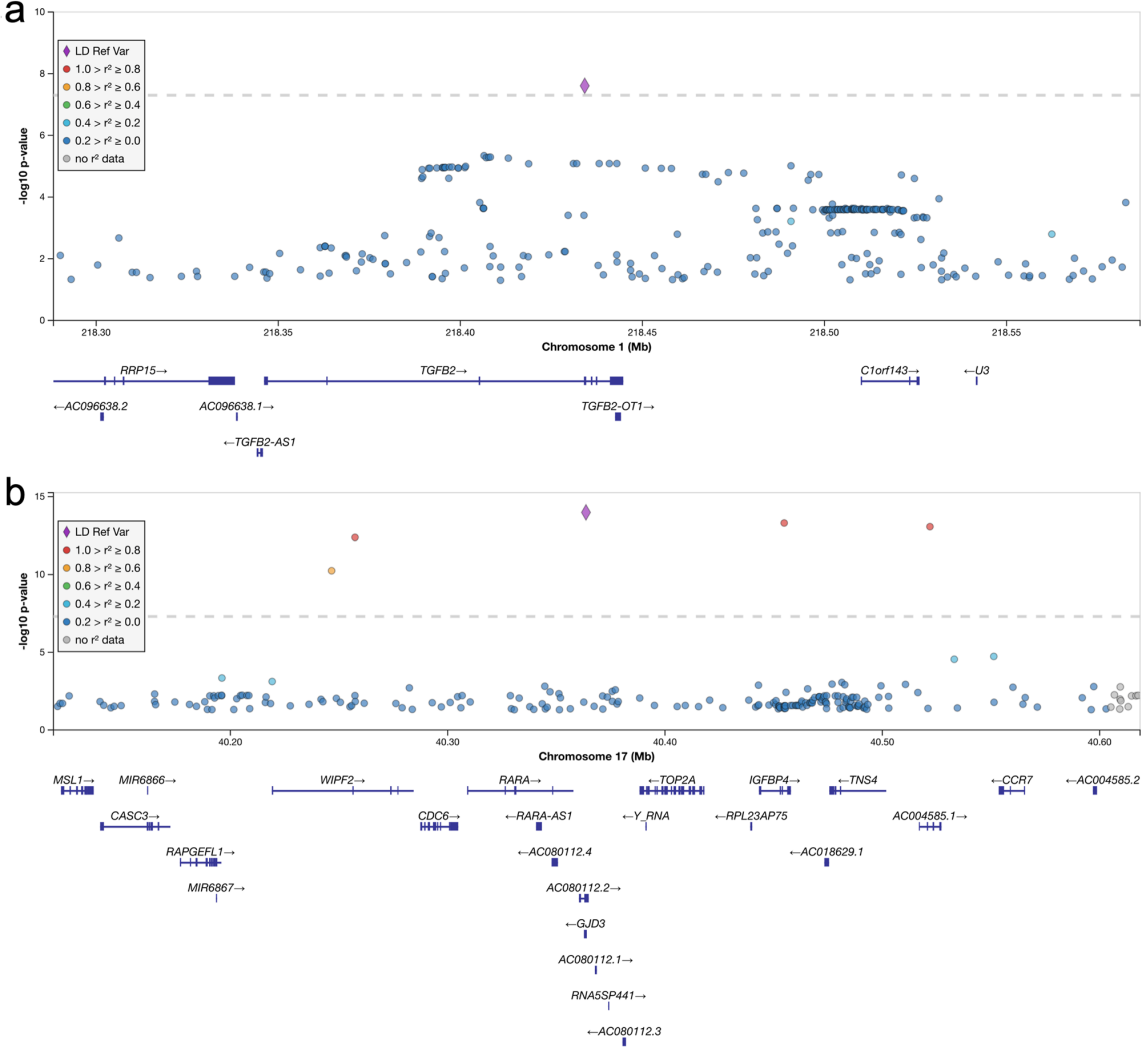
Volcano plots of *TGFB2* and *GJD3* lead SNP PheWASs. The disease event associations of the phenome-wide scan of rs192335285-T (*TGFB2*) in **a** and rs201955556-T (*GJD3*) in **b** are plotted with their −log_10_-transformed *P* values and the associated effect (*β*) as the logarithm of the odds ratio along the vertical and horizontal axes, respectively. The categories of the 1706 analyzed disease events are indicated according to the legend right of the figure. Phenome-wide significant (*P* < 6.1 × 10^−7^) disease endpoint associations have been annotated with their disease endpoint name.

We observed that a synonymous variant (rs192335285-T; MAF in Finns = 2.5 × 10^−3^; Finnish Enrichment = 56.133; PIP = 0.74) in the fourth exon of the loss-of-function intolerant (pLI = 1 in gnomAD) *TGFB2* was associated with a higher risk for VV (OR = 2.12 [1.65–2.85], *P* = 2.5 × 10^−8^). rs192335285-T is predicted to be deleterious (CADD score = 13.03) and creates a new splice-site with a high probability (SpliceAI delta score = 0.84 [donor-gain]) at a site that is highly conserved (GERP = −1.65). Further evidence of the impact of rs192335285-T on splicing comes from a previous cDNA clones-based study of *TGFB2* that showed the existence of two proteins formed by alternative splicing at the variant site^36^. For these reasons, rs192335285-T has a high probability of being a loss-of-function variant. In addition, rs192335285-T had no variants in linkage disequilibrium (Fig. 4a) and had a posterior inclusion probability (PIP) of 0.49, which support the notion that rs201955556-T is driving the association with VV risk.

In the phenome-wide scan, we observed that rs192335285-T was highly associated with the risk of hernia (OR = 2.02 [1.65–2.46], *P* = 5.6 × 10^−12^), diaphragmatic hernia (OR = 3.58 [2.31–5.55], *P* = 1.2 × 10^−8^) and inguinal hernia (OR = 1.91 [1.48–2.45], *P* = 5.4 × 10^−7^) in addition to VV and directly VV-related endpoints (Fig. 3a and the *rs192335285–TGFB2* tab in Supplementary Data 4). Moreover, haploinsufficiency in *TGFB2* causes thoracic aortic aneurysms^37,38^ and we replicate this result by observing an association between aortic aneurysm risk and rs192335285-T (OR = 2.53 [1.43–4.45], *P* = 1.3 × 10^−3^; 2825 cases). The *TGFB2* variant was nominally associated (*P* < 0.05) with 309 disease endpoints in line with the diversity of cellular processes that the TGF-β signaling pathway regulates, which includes growth, differentiation, and apoptosis.

As per the observation that a member of the TGF-β signaling pathway is associated with VV risk we consequently checked if any other genes in the TGF-β pathway^39^ were associated with VV risk. The common intronic variant in *TGFBR3* (rs6662887-T; MAF in Finns = 0.297; Finnish Enrichment = 0.917; PIP = 0.74), newly identified in our analyses, was associated with a lower risk for VV (OR = 0.91 [0.89–0.94], *P* = 4.0 × 10^−9^,). Subsequently, we checked if any of the TGF-β pathway genes were associated with VV risk on a gene-level. The TGF-β receptor 2 (*TGFBR2*) was also associated with VV risk because protein-truncating variants in the gene were associated with a higher risk for VV in lower extremities (SKAT-O test, *β* = 0.13, *P* = 6.7 × 10^−6^ based on 281,852 UK Biobank exomes^40^ [genebass.org]). Together these findings implicate the TGF-β signaling pathway more broadly involved in VV etiopathology.

### *GJD3* and connexins in VV

We observed an association between a missense variant (rs201955556-T; PIP = 0.45) in the only exon of Gap Junction Protein Delta 3 (*GJD3*) and a lower risk for VV (OR = 0.62 [0.55–0.70], *P* = 1.0 × 10^−14^). *GJD3* encodes a member of the large family of connexins which are membrane proteins that form intercellular channels and gap junctions that enable low molecular weight transport between cells^41^, and have been shown to play important roles in inflammation, wound repair and thrombosis^42–46^. No prior disease associations with *GJD3* have been reported, but the only other previously reported missense variant in the gene was associated with lower sex hormone binding globulin (SHBG) levels^47^ and lower platelet distribution width (PDW)^48^. Moreover, in burden tests of *GJD3* missense variants were associated with lower levels of SHBG and urate and lymphocyte percentages^40^.

The rs201955556-T variant is predicted to be deleterious (SIFT prediction = deleterious; Polyphen = probably damaging; CADD score = 29.1) and changes the amino acid at position 59 of the encoded gap junction protein (HGVS p.Pro59Thr) affecting one of the two of its extracellular domains. In addition, rs201955556-T was the only protein-coding variant and had the highest PIP = 0.450 in a credible set with only five other variants (Supplementary Data 2). Also, the *GJD3* locus plot supports the notion that rs201955556-T is independently associated with VV risk (Fig. 4b). In the phenome-wide scan rs201955556-T was associated (*P* < 6.1 × 10^−7^) with a lower risk of varicose veins endpoints only which suggests a highly specific etiopathology of *GJD3* on VV (Fig. 3b and the *rs201955556*–*GJD3* tab in Supplementary Data 4). Given the rarity of rs201955556-T in non-Finnish data sets (UK Biobank exomes allele count = 1) as well as the absence of *GJD3* PTVs in FinnGen and other biobank data sets, we were not able to replicate the association between *GJD3* and VV risk. Nevertheless, a lower risk of varicose vein surgery (*β* = −3.9 × 10^−3^, *P* = 2.2 × 10^−3^) is the top disease endpoint association in a *GJD3* missense burden analysis of 281,852 UK Biobank exomes^40^, albeit this association was not significant after multiple testing correction.

We investigated whether the role of connexin genes in VV etiopathology extends beyond *GJD3*. We examined which protein-coding variants in 21 connexin genes associated with VV risk. Across the 47 protein-coding variants (MAF > 0.001) in 17 connexin in the FinnGen data, we discovered no other associations with VV risk (all *P* > 1.8 × 10^−3^; Supplementary Data 7), suggesting a *GJD3* specific effect for VV. Upon examination of other FinnGen endpoints, we found a frameshift variant in *GJB2* (rs80338939-A, Finnish enrichment = 1.416), reported in ClinVar as pathogenic for nonsyndromic hearing loss and deafness, associated with deaf mutism (OR = 249.9 [34.3–1819.6], *P* = 5.0 × 10^−8^; 80 cases), hence replicating a known connexin disease association.

## Discussion

We set out to further our understanding of the biology of VV by carrying out a genome-wide scan of 13,541,466 genetic variants in 207,055 Finns leveraging the detailed genotype data coupled with longitudinal national health registries available in the FinnGen dataset (fifth data release). The key research motivations of the current study were, first, to identify new potential intervention targets for VV with the help of novel loci and protein-coding variants associated with VV risk and, second, to investigate if the sex-based differences in VV prevalence could be in part explained using VV genetics.

We observed 50 genetic loci associated with VV risk including 29 loci newly reported to associate with VV in our analyses. Of the 29 novel loci 27 are autosomal, one of which was associated with VV risk exclusively in females and two that implicate the TGF-β as a novel pathway of VV, as well as two that are located on the X chromosome. The lead variants of two novel loci (*TGFB2* and *GJD3*) were over 56-fold Finnish-enriched low-frequency coding variants with a large associated effect(|*β*| > 0.47) on VV risk. The missense variant rs201955556-T in *GDJ3* was associated with a substantially lower VV risk and showed no pleiotropy in a phenome-wide scan of >1700 disease endpoints highlighting the potential of connexin modulation as a therapy for VV. Overall our results support the use of samples from isolated populations for increased statistical power to, first, gain insight into the etiology of complex diseases^21–28^ such as VV and, second, in finding therapeutic target genes.

Despite half the sample size in FinnGen compared to the largest VV GWAS to date^17^, more than half (29 out of 50) of the loci identified in our analyses are previously unreported for VV. We attribute some of the new locus discovery to the inclusion of sex-specific and X-chromosomal analyses in the VV GWAS which to the best of our knowledge we are the first to do and potentially to the more precise and conservative case ascertainment which is based solely on longitudinal health records. The higher share of Finnish-enriched and coding low-frequency variants among our novel loci compared to known loci observed in our analysis nevertheless suggests that the gain in statistical power is primarily attributable to the unique genetic characteristics of the Finnish population.

Exemplifying the potential of finding putative drug targets using coding-variant associations in isolated populations, among our novel VV loci, we identified the 69-fold Finnish-enriched missense variant rs201955556-T in *GJD3* that was associated with 38% lower odds of VV. *GJD3* encodes a member of the connexin family of proteins which are linked to wound healing through various mechanisms^44–46^ and have been subject to therapy development^49^. *GJD3* creates subunits of connexons which are transmembrane channels which have the attractive property of being accessible to molecular targeting because they reside on the surface of cells. This fact together with the association between the rs201955556-T and lower VV risk makes *GJD3* an especially attractive potential intervention target gene. Further, as far as potential drug target safety goes, we observed only associations between rs201955556-T and VV risk in our phenome-wide analysis of 1706 disease endpoints. These findings echo the discovery of another VV-associated gene that encodes a transmembrane channel, the mechanosensitive *PIEZO1*, where both VV risk-increasing loss-of-function and a risk-decreasing missense variants associated with VV suggest therapeutic pathways for VV^50,51^.

When reflecting on the drug target potential of *GJD3* functional validation is warranted. However, the missense variant rs201955556-T is probably damaging (CADD score = 29.1) and affects the extracellular domains of the connexin hemichannel. This could impact how well the hemichannels dock to one another when forming a gap junction between two cells and in turn influence the molecular transport between cells. Several proteins of the connexin family are known to affect platelet function^52,53^, which is critical for wound repair and thrombosis. A previously reported GWAS association between another missense variant in *GJD3* and lower platelet distribution width supports the notion that *GJD3* affects VV pathology through platelet function. Furthermore, this same missense variant has also been associated with lower SHBG levels which are linked to blood coagulation through the anticoagulant protein S^54^ and thrombosis through epidemiological evidence^55–57^.

Our newly identified observations that *TGFB2* and *TGFBR3* loci are associated with VV risk pinpoint TGF-β as a pathway of VV. Previous non-genetic studies support TGF-β pathway’s involvement in VV pathology through regulating the remodeling of the extracellular matrix of vascular smooth muscle cells^58–60^. The lead variant in *TGFB2* was over 56-fold enriched in Finns compared to NFEEs and is predicted to create a new transcript splice-site and thus potentially acts as a loss-of-function variant. Although the carriers of rs192335285-T had substantially higher risk of VV than non-carriers, *TGFB2* is unlikely to be suitable as a drug target gene for VV due to its extensive pleiotropy that we observed in our comprehensive phenome-wide analysis in the FinnGen Study.

While the unique Finnish population genetics is an asset of our study it also poses limitations. Our study lacks the replication of our key results as the Finnish-specific variants, including rs201955556-T in *GJD3*, which are not available or are poorly imputed in UK Biobank, Biobank Japan and Million Veterans Program data sets. Similarly, replication was not possible either via *GJD3* loss-of-function variants in these data sets or in the FinnGen data given the rarity of such variants. Additionally, we would also need population-specific functional data to ascertain the mechanism of how rs201955556-T influences VV pathology. Nevertheless, we replicated with same direction effects and *P* < 1.3 × 10^−3^ (two-tailed *P* value threshold Bonferroni-corrected for 37 tests) 33 out of 37 sex-shared lead variants common for both FinnGen and UK Biobank data^17^, which limits the possibility of false positive findings.

Although some conflicting evidence exists^2,9–12^, many epidemiological studies have found VV is more often diagnosed in females than males^3–8^. Given the higher female risk for diagnosed VV observed in the present study, we additionally examined the evidence for sex differences in VV genetics in the FinnGen data. Our results point to high similarity in genetic effects and variance between males and females which suggests little overall contribution from genetic factors to explain the sex differences in VV. These observations echo the findings for other complex traits where sex differences in genetic architecture have been found to be in general small in magnitude^61–63^. Our sex-specific genetic analyses nevertheless identified one female-specific locus near *ERG*. Such a finding highlights the value of sex-stratified assessment of genetic risk factors to uncover genetic loci and biology that could be masked in sex-agnostic analyses^61^.

In summary, we identified 27 autosomal and two X-chromosomal novel loci associated with VV risk. We established the high probability of the TGF-β pathway’s involvement in VV etiopathology, show limited evidence for sex differences in VV genetics and uncovered *GJD3* as a possible drug target gene for VV. These findings underscore the utility of genetic studies in isolated populations in uncovering genetic associations and putative therapeutic targets for complex diseases.

## Methods

### Ethics statement

All FinnGen participants gave written informed study-specific consent. Patients and control subjects in the FinnGen Study provided informed consent for biobank research, based on the Finnish Biobank Act. Alternatively, older Finnish research cohorts, collected prior to the start of the FinnGen Study (August 2017), were collected based on study-specific consents and later transferred to the Finnish biobanks after approval by Fimea, the National Supervisory Authority for Welfare and Health. Recruitment protocols followed the biobank protocols approved by Fimea. The Coordinating Ethics Committee of the Hospital District of Helsinki and Uusimaa (HUS) approved the FinnGen Study protocol No. HUS/990/2017.

The FinnGen project is approved by Finnish Institute for Health and Welfare (THL), approval number THL/2031/6.02.00/2017, amendments THL/1101/5.05.00/2017, THL/341/6.02.00/2018, THL/2222/6.02.00/2018, THL/283/6.02.00/2019), Digital and population data service agency VRK43431/2017-3, VRK/6909/2018-3, the Social Insurance Institution (KELA) KELA 58/522/2017, KELA 131/522/2018, KELA 70/522/2019 and Statistics Finland TK-53-1041-17.

The Biobank Access Decisions for FinnGen Study samples and data utilized in FinnGen Data Freeze 4 include: THL Biobank BB2017_55, BB2017_111, BB2018_19, BB_2018_34, BB_2018_67, BB2018_71, BB2019_7 Finnish Red Cross Blood Service Biobank 7.12.2017, Helsinki Biobank HUS/359/2017, Auria Biobank AB17-5154, Biobank Borealis of Northern Finland_2017_1013, Biobank of Eastern Finland 1186/2018, Finnish Clinical Biobank Tampere MH0004, Central Finland Biobank 1-2017, and Terveystalo Biobank STB 2018001.

UK Biobank obtained ethical approval from the North West Multi-Centre Research Ethics Committee (MREC) (11/NW/0382) to collect and disseminate data and samples from participants and all participants gave informed consent for their genotype data for research use. UK Biobank VV GWAS summary statistics used for analyses carried out in this study came from a study by Ahmed et al.^17^, which was conducted under UK Biobank study no. 22572.

No informed written consent was obtained from GTEx participants as they were deceased when their tissue specimens were collected. For details on the authorization of GTEx as a study please see the Consenting Donors section in the Materials and methods section of Carithers et al.^64^.

### Discovery cohort

The FinnGen Study data release 5 (R5) contains biobank data and national health registry data for 218,792 individuals. All FinnGen Study participants were of Finnish descent. Information on diagnoses in the FinnGen data were collected and confirmed by examining national healthcare registries and recorded using the *International Classification of Diseases* [ICD] revisions 8–10. Purchase information on prescription drugs since 1995 were obtained from the Finnish social insurance institution (KELA) reimbursement records and coded using the *Anatomical Therapeutic Chemical* [ATC] classification). All FinnGen Study participants’ healthcare registry information were followed from the year 1969 up until 31.12.2018. Cancer diagnoses and causes of death were obtained from their respective national registries. The clinical expert groups of the FinnGen Study have defined disease events using ICD and ATC codes. For a complete list of the considered clinical endpoints and corresponding ICD and ATC codes, see the link: www.finngen.fi/en/researchers/clinical-endpoints. The endpoints can also be browsed at r5.risteys.finngen.fi.

For the VV analyses, the FinnGen data consisted of 17,027 (13,045 females, 3982 males) VV cases and 190,028 vein and lymphatic disease-free individuals as controls of VV. Case individuals were those who during follow-up had received a diagnosis in the following ICD code categories: I83 (ICD-10) and 454 (ICD-9 and ICD-8). The controls excluded all individuals with a group of diagnoses that includes diseases of veins, lymphatic vessels and lymph nodes (see Supplementary Data 8 or r5.risteys.finngen.fi/phenocode/I9_VARICVE for the ICD-based definition). The share of cases that received a VV diagnosis by the ICD codes: I83.9, 454, and not a more severe diagnosis by the ICD-10 codes: I83.0 or I83.1, was 91.3% and 79.5% among females and males respectively. Yet the fact that rs11121615-T (*CASZ1* lead SNP) is the most significantly associated variant in the FinnGen data, indicates that the VV cases are not at least substantially confounded by the least severe form of VV, i.e. telangiectasia or reticular veins (C1 on the CEAP classification system^65^), as earlier studies have observed this *CASZ1* variant is not associated with these forms of venous disease^66^. The average age at the end of follow-up for VV controls was 12.2 and 10.7 years higher than the sex-specific VV diagnosis age for females and males (Supplementary Data 9).

### Genotyping and quality control

237,417 FinnGen Study samples were genotyped using various Illumina arrays and the custom AxiomGT1 Affymetrix array (www.finngen.fi/en/researchers/genotyping) at the Thermo Fisher genotyping service facility (San Diego, USA). The individuals not genotyped on the AxiomGT1 Affymetrix array came from 29 merged datasets from the Finrisk, Botnia, H2000/2011, Generisk and Psychiatric Family Collections, Auria, Borealis, DIME, FT17, HBP, Iddmgen, VPU and YA cohorts (Supplementary Data 10).

The haplotype estimation for FinnGen Study samples we carried out with Eagle2^67^, v2.3.5 (alkesgroup.broadinstitute.org/Eagle/). Subsequently, we imputed missing genotypes of FinnGen Study participants with Beagle^68^, version 4.1 (faculty.washington.edu/browning/beagle/beagle.html), and a Finnish ancestry-specific reference panel consisting of 3775 deep-coverage (25–30×) whole-genome sequences from the SISu project (Sequencing Initiative Suomi, www.sisuproject.fi)^69^. The number of samples before and after genotype imputation, as well as the number of chip-genotyped variants for each genotyping batch are listed in Supplementary Data 10. The genotype imputation protocol for FinnGen data is described by Pärn et al.^70^, v3.0 (10.17504/protocols.io.xbgfijw).

### Sample and variant-level quality control

The FinnGen Study genotypes first underwent basic variant and sample-level QC. Variants with a call rate less than 97%, and samples with genotyping missingness greater than 5% or with ambiguous sex were removed. Sample mix-ups and extraneous duplicate copies of lower genotyping quality we removed manually. Then we excluded individuals with an ambiguous genotype-determined sex (*F*-score >0.3), a genotyping success rate below 95%, excess heterozygosity (more than 4 standard deviation units from the mean or non-European ancestry), outside the population structure as specified by multidimensional scaling (maximum of five iterations) or by the first two principal components (beyond 4 standard deviation units from the mean with a maximum of five iterations) or contaminated samples (pihat linkage ≥0.1 with at least 14 samples). We iterated through these steps until all samples met all the criteria.

On the FinnGen data we performed population outlier detection using principal component analysis (PCA) and a Bayesian algorithm using 41,678 independent and common variants with a high genotype probability and low missingness. These variants met the following criteria: located in an autosomal chromosome, an IMPUTE2 genotype information score ≥0.95, missingness ≤0.01 (according to GP [genotype probability]), and a MAF ≥ 0.05. Next the remaining variants underwent LD-pruning with a window size of 500 kb, a step size of 50 kb and a *r*^2^ filter <0.1. We took the genotypes of the 41,678 variants from the 1000 Genomes Project^71^ (1000G) and merged them with the corresponding FinnGen genotype data. Then, because of the outlier detection, we removed 5520 samples of which 3138 were FinnGen Study samples. Supplementary Fig. 2 shows the scatter plots of the genetic components of each sample projected onto the first three principal components. This routine successfully detected all the 1000G samples with non-European and Southern European ancestry but failed to exclude all 1000G samples with Western European origin. The cluster of Western Europeans classified as Finns was too small to perform a second round of population outlier detection, using the PCA and Bayesian algorithm routine, without detecting substructures of the Finnish population. Therefore, we performed another PCA on the remaining FinnGen Study samples and projected them as well as the European and Finnish 1000G genotypes onto this new set of three principal component axes (Supplementary Fig. 3). Next, we calculated the centroids of each sample cluster and the squared Mahalanobis distances of the FinnGen samples to the centroid of each cluster. Since the squared Mahalanobis distance is a sum of variables with unit variance, we can see it as a sum of three independent variables and thus generate a *χ*^2^ probability distribution with 3 degrees of freedom. In this way, for each sample, we were able to calculate its probability of belonging to each cluster. As a result, we removed 538 FinnGen samples considered outliers due to a lower than a 0.95 probability of belonging to the FinnGen sample cluster (Supplementary Fig. 3).

To maximize the number of unrelated samples used in our association tests we first determined all the pairs of FinnGen samples up to the second degree. The distribution of kinship values of the FinnGen samples is shown in Supplementary Fig. 4. Thereafter, we used two algorithms from the *network* Python package (networkx.github.io/) to flag samples up to second degree kinship in the remaining FinnGen Study samples; *greedy*, to remove the highest degree node from the network of relations until no more links in the network remain, and *native* applied on each subgraph of the network. These two algorithms separated the samples into three sets: 156,977 unrelated samples with Finnish ancestry, 61,980 non-duplicate samples with Finnish ancestry but who are related to the samples in the first set, and 5780 samples who are either of non-Finnish ancestry, twins or duplicates related to other samples which we then excluded. We carried out a PCA on the 156,977 unrelated samples after which we projected the 61,980 related samples in the second sample set onto the same multidimensional space (Supplementary Fig. 5) yielding population covariates for 218,957 samples. Additionally, we excluded 165 samples with missing minimum phenotype information or a mismatch between imputed sex and the reported sex in the registry data. Thus, the FinnGen cohort we used for analyses consisted of 218,792 samples in total.

### Statistics and reproducibility

First we sought to identify associations between single variants and VV risk in a genome-wide manner. Our model was additive and in the sex-combined analyses we included age, sex, genotyping batch, ten principal components of ancestry and the kinship matrix as fixed-effects covariates. The odds ratios for VV and all the other disease outcomes were estimated using SAIGE^19^, version 0.35.8.8 (www.github.com/weizhouUMICH/SAIGE/releases/tag/0.35.8.8). To avoid convergence issues, a genotyping batch was included as a covariate for an endpoint if the batch contained at least ten cases and controls. One genotyping batch was not included as a covariate in the model to avoid the saturation of covariate values. We excluded the AxiomGT1_b16 genotyping batch (Supplementary Data 10) as it was not enriched for any disease endpoint.

For calculating the kinship matrix, we used a genotype dataset in which we, first, set genotypes with a GP ≥ 0.95 as missing, second, included only variants with an IMPUTE2 genotype information score > 0.95 and, third, used variants with ≤0.03 genotype missingness and a MAF ≥ 0.01. Subsequently, the remaining variants were LD-pruned with a 1 Mb window and an *r*^2^ < 0.1 threshold which resulted in a set of 58,702 well-imputed and non-rare variants for calculating the kinship matrix. The SAIGE options in place for computing the kinship matrix were: LOCO = false, numMarkers = 30, traceCVcutoff = 0.0025 and ratioCVcutoff = 0.001.

We also carried out a GWAS of VV risk for males (3982 cases and 86,041 controls) and females (13,045 cases vs 103,987 controls) separately using SAIGE and with a regression model with the same kind of fixed-effects covariates as for sex-combined analysis except for sex. We considered the genome-wide association significance threshold of a two-sided *P* value of <5.0 × 10^−8^ to be significant. In our scan, we only considered variants with a MAF of at least 0.001 to account for adequate statistical power. Independent genome-wide significant loci were defined as 2 Mb sized windows centered around each lead variant, i.e., the variant with the smallest *P* value in each region. The independent loci that we obtained by repeatedly equating the lead variant as the statistically most significant variant for each chromosome and pulling out the variants that are within a 2 Mb window until no genome-wide significant variants were left. Novel loci were those that were more than 1 Mb from previously reported lead variants^16–20^. The variant type annotation we obtained using the Ensembl Variant Effect Predictor^72^. To assess the statistical independence of the variant associations with VV risk, we determined the 95% credible sets of variants overlapping all loci outside the HLA region (chr6:28,510,120–33,480,577 [GRCh38]) (Supplementary Data 2). The credible sets were determined using SuSiE^33^ (stephenslab.github.io/susie-paper). The Finnish allele enrichments compared to NFEEs we computed from the ratio of the allele frequencies in Finns and NFEEs in the gnomAD database^30^, v2.1.1.

We screened the lead variants broadly for modified risk of 1706 disease endpoints with a minimum of 500 cases in the FinnGen data. We regarded a two-sided *P* value below 6.1 × 10^−7^ (Bonferroni-corrected threshold for 1706 traits and 48 lead variants [81,888 tests] outside the HLA region [chr6:28,510,120–33,480,577]) to be statistically significant. To replicate the PheWAS findings, for each independent VV locus we conducted genetic colocalization tests between VV and the corresponding phenome-wide significantly associated trait(s). We used the *coloc.abf()* function from the *coloc* R-package^35^, version 5.1.0 (cran.r-project.org/web/packages/coloc/index.html) to compute the posterior probabilities for the five hypotheses: H0: no association, H1: association to trait VV risk only, H2: association to trait other trait only, H3: association to both VV risk and other trait with distinct causal variants and H4: association to both VV risk and other trait with a shared causal variant. We used the default values for the prior probabilities p1 (prior probability that an SNP is associated with trait 1: 1 × 10^−4^), p2 (prior probability that an SNP is associated with trait 2: 1 × 10^−4^) and p12 (prior probability that an SNP is associated with both traits: 1 × 10^−5^). We regarded a posterior probability above 0.8 for colocalization with the same causal variant (PP.H4 > 0.8) as replicating. The details of computing the associations in the FinnGen data are described in the Supporting information.

We tested the genetic correlations between VV and traits that were phenome-wide significantly associated (two-sided *P* < 6.1 × 10^−7^) with the lead variants from the VV GWAS. The traits also had to have non-zero heritability estimates using LDSC^31,32^, v1.0.1 (github.com/bulik/ldsc). First, we ran a logistic model genome-wide association using PLINK^73^, v1.90b6.20, and v2.00a3LM (www.cog-genomics.org/plink), and then used LDSC^31,32^ to estimate the variant-based heritability of VV. To avoid deflating the single-variant heritability estimates when computing heritability estimates, the single-variant association analysis was performed without a kinship matrix.

We carried out colocalization analyses to test whether the VV GWAS association signals from the present study and the eQTL association signals from VV etiopathologically relevant GTEx V8 tissues (adipose, arterial and nerve tissues) had a shared causal genetic variant at loci of size 2 Mb centered at the GWAS lead variants. We chose these tissues based on prior evidence for their relevance in earlier studies. Ahmed et al.^17^ found significant enrichments in sex-shared tissue types for blood vessel, nerve and adipose tissue. Furthermore, Fukaya et al.^16^ point to adipose tissue in VV genetics. We used the *coloc.abf()* function from the *coloc* R-package^35^, version 5.1.0 (cran.r-project.org/web/packages/coloc/index.html), to compute the posterior probabilities for the five hypotheses: H0: no association, H1: association with trait VV risk only, H2: association with trait gene expression levels only, H3: association with both VV risk and gene expression levels with distinct causal variants and H4: association with both VV risk and gene expression levels with a shared causal variant. We used the default values for the prior probabilities p1 (prior probability that an SNP is associated with trait 1: 1 × 10^−4^), p2 (prior probability that an SNP is associated with trait 2: 1 × 10^−4^) and p12 (prior probability that an SNP is associated with both traits: 1 × 10^−5^).

### Reporting summary

Further information on research design is available in the Nature Portfolio Reporting Summary linked to this article.

## Supplementary information

Supplementary Information

Description of Additional Supplementary Files

Supplementary Data 1

Supplementary Data 2

Supplementary Data 3

Supplementary Data 4

Supplementary Data 5

Supplementary Data 6

Supplementary Data 7

Supplementary Data 8

Supplementary Data 9

Supplementary Data 10

Reporting Summary

## Data Availability

The genome-wide variant summary statistics for all the sex-combined disease event phenotypes in FinnGen are downloadable from console.cloud.google.com/storage/browser/finngen-public-data-r5/summary_stats and those of VV are indicated with the phenotype code *I9_VARICVE*. The sex-stratified GWAS summary statistics are available in GWAS catalog. We used the VV GWAS summary statistics calculated in UK Biobank by Ahmed et al.^17^, which are accessible at 10.5287/bodleian:8J26woZQg. The GTEx^34^ V8 eQTL variant summary statistics are publicly available at gtexportal.org/home/datasets. Source data underlying Figs. 2b and 4 are presented in Supplementary Data 3 and 4, respectively.

## Code availability

The complete genotype imputation protocol for FinnGen data is described by Pärn et al.^70^ at 10.17504/protocols.io.xbgfijw. Pipelines for performing quality control and genotype imputation of the FinnGen data^74^ are available at 10.5281/zenodo.7329050. Furthermore, the PCA and kinship pipeline^75^ is available at 10.5281/zenodo.7329714. Pipelines for the variant association analyses^76^ and genetic finemapping^77^ with FinnGen data are available at 10.5281/zenodo.7329765 and 10.5281/zenodo.7329773, respectively.
