# Supplementary material for: Genome-wide association study of varicose veins identifies a protective missense variant in GJD3 enriched in the Finnish population
Source: Commun Biol. 2023 Jan 18;6:71. doi: 10.1038/s42003-022-04285-w (PMC9849365; doi:10.1038/s42003-022-04285-w)
Supplement: Supplementary file 2 — Description of Additional Supplementary Files [file 42003_2022_4285_MOESM2_ESM.pdf]

Description of Additional Supplementary Files

Filename: Supplementary Data 1

Description: Lead variants summary statistics of the 50 observed genome-wide significantly associated (P < 5.0e-8) genetic loci along with their UK Biobank replication test statistics.

Filename: Supplementary Data 2

Description: Summary statistics of the 95% credible sets within the genome-wide significantly associated non-HLA region varicose veins loci observed in the FinnGen Study.

Filename: Supplementary Data 3

Description: Sex-specific associated effect statistics for the varicose veins lead variants identified in the corresponding sex-combined GWAS.

Filename: Supplementary Data 4

Description: Association statistics from the FinnGen Study phenome-wide scans of the varicose veins lead variants outside the HLA region.

Filename: Supplementary Data 5

Summary of Genetic Correlation Results between varicose veins (I9_VARICVE) and 31 distinct phenome-wide significantly associated phenotypes.

Filename: Supplementary Data 6

Description: Colocalisation statistics between gene expression level associations and varicose veins risk association statistics in varicose veins associated loci observed in the FinnGen data.

Filename: Supplementary Data 6

Description: A list of the 21 human connexins and their corresponding genes together with association statistics between protein-coding variants (> 1e-3 MAF) in these genes and their varicose veins risk in the FinnGen data.

Filename: Supplementary Data 7

Description: ICD-code-based case and control definitions for the varicose veins phenotype definition used in the FinnGen Study.

Filename: Supplementary Data 8

Description: Breakdown of varicose veins status by sex and age at death or end of

follow-up, baseline age and diagnosis age.

Filename: Supplementary Data 9

Description: Breakdown of the no. of FinnGen Study samples of the fifth data release (R5)

per genotyping batch before the quality control (QC) prior to the individuals' missing

genotypes have been imputed, and ultimately the amount of imputed samples.

Filename: Supplementary Data 10

Breakdown of the no. of FinnGen Study samples of the fifth data release (R5) per

genotyping batch before the quality control (QC) prior to the individuals' missing

genotypes have been imputed, and ultimately the amount of imputed samples.
