# Supplementary material for: Genome-wide association study of varicose veins identifies a protective missense variant in GJD3 enriched in the Finnish population
Source: Commun Biol. 2023 Jan 18;6:71. doi: 10.1038/s42003-022-04285-w (PMC9849365; doi:10.1038/s42003-022-04285-w)
Supplement: Supplementary file 13 — Reporting Summary [file 42003_2022_4285_MOESM13_ESM.pdf]

nature portfolio

Corresponding author(s): Taru Tukiainen

Last updated by author(s): Nov 3, 2022

# Reporting Summary

Nature Portfolio wishes to improve the reproducibility of the work that we publish. This form provides structure for consistency and transparency in reporting. For further information on Nature Portfolio policies, see our Editorial Policies and the Editorial Policy Checklist.

## Statistics

For all statistical analyses, confirm that the following items are present in the figure legend, table legend, main text, or Methods section.

| n/a | Confirmed | |
|---|---|---|
| ☐ | ☒ | The exact sample size (*n*) for each experimental group/condition, given as a discrete number and unit of measurement |
| ☐ | ☒ | A statement on whether measurements were taken from distinct samples or whether the same sample was measured repeatedly |
| ☐ | ☒ | The statistical test(s) used AND whether they are one- or two-sided<br>*Only common tests should be described solely by name; describe more complex techniques in the Methods section.* |
| ☐ | ☒ | A description of all covariates tested |
| ☐ | ☒ | A description of any assumptions or corrections, such as tests of normality and adjustment for multiple comparisons |
| ☐ | ☒ | A full description of the statistical parameters including central tendency (e.g. means) or other basic estimates (e.g. regression coefficient) AND variation (e.g. standard deviation) or associated estimates of uncertainty (e.g. confidence intervals) |
| ☐ | ☒ | For null hypothesis testing, the test statistic (e.g. *F*, *t*, *r*) with confidence intervals, effect sizes, degrees of freedom and *P* value noted<br>*Give P values as exact values whenever suitable.* |
| ☒ | ☐ | For Bayesian analysis, information on the choice of priors and Markov chain Monte Carlo settings |
| ☒ | ☐ | For hierarchical and complex designs, identification of the appropriate level for tests and full reporting of outcomes |
| ☐ | ☒ | Estimates of effect sizes (e.g. Cohen's *d*, Pearson's *r*), indicating how they were calculated |

*Our web collection on statistics for biologists contains articles on many of the points above.*

## Software and code

Policy information about availability of computer code

Data collection

No software was used for data collection.

Data analysis

We used following pieces of software for genotype imputation, handling the genetic and phenotypic data and the analyses:
Cromwell 61
Plink v1.90b6.20
BCFtools versions 1.7 and 1.9
Eagle version 2.3.5
Beagle 4.1 (08Jun17.d8b)
Coloc R-package, version 5.1.0
SAIGE, version 0.35.8.8
Ensembl Variant Effect Predictor
SuSiE
LDScore regression
The full genotype imputation protocol for FinnGen is described at dx.doi.org/10.17504/protocols.io.xbgfijw. Pipelines for performing quality control and genotype imputation of FinnGen data are available at https://github.com/FINNGEN. The same repository also contains code for conducting association analyses genome- and phenome-widely, and genetic finemapping with FinnGen data.

For manuscripts utilizing custom algorithms or software that are central to the research but not yet described in published literature, software must be made available to editors and reviewers. We strongly encourage code deposition in a community repository (e.g. GitHub). See the Nature Portfolio guidelines for submitting code & software for further information.

# Data

Policy information about availability of data

All manuscripts must include a data availability statement. This statement should provide the following information, where applicable:

- Accession codes, unique identifiers, or web links for publicly available datasets
- A description of any restrictions on data availability
- For clinical datasets or third party data, please ensure that the statement adheres to our policy

The Finnish biobank data can be accessed through the Fingenious® services (web link: https://site.fingenious.fi/en/, email: contact@finbb.fi) managed by FINBB. The complete set of results described in the main text are located in the Supplementary Data 1-10 files.The genome-wide variant summary statistics for all the sex-combined disease event phenotypes in FinnGen are downloadable from https://console.cloud.google.com/storage/browser/finngen-public-data-r5/summary_stats/ and those of varicose veins (VV) are indicated with the phenotype code "I9_VARICVE". We used the VV GWAS summary statistics calculated in UK Biobank by Ahmed et al. (doi.org/10.1038/s41467-022-30765-y) which are accessible at https://doi.org/10.5287/bodleian:8J26woZQg. The GTEx V8 eQTL variant summary statistics are publicly available at https://gtexportal.org/home/datasets.

# Human research participants

Policy information about studies involving human research participants and Sex and Gender in Research.

**Reporting on sex and gender**

In addition to sex-combined analyses, we carried out sex-stratified genome-wide analyses of VV with the FinnGen data and report and compare their genome-wide significant variant associations in the paper. FinnGen samples with ambiguous sex, e.g., mismatch between reported sex and sex inferred from genotype data, were removed during the sample-level genotype quality control.

**Population characteristics**

This study included participants from the fifth data release of FinnGen. All individuals used in this study were of Finnish ancestry. The FinnGen VV dataset consisted of 17,027 (13,045 females, 3,982 males) VV cases and 190,028 vein and lymphatic disease-free individuals as controls of VV. The mean age for first VV disease event was 45.0 years and 43.6 and 49.6 years for females and men respectively.

The key characteristics of the FinnGen study participants are described in the main text of the manuscript, Table 1, Figure 2a and Supplementary Data 9.

The population characteristics of UK Biobank samples used to compute the VV UK Biobank variant association summary statistics are described in the Population and phenotype definition section of publication by Ahmed et al. (doi.org/10.1038/s41467-022-30765-y)

**Recruitment**

FinnGen consists of a random sample of subjects from Finnish population-based cohorts and clinical biobanks. A proportion of FinnGen was ascertained through hospital biobanks and disease-based collections.

UK Biobank recruited circa 500,000 participants between ages 40 and 69 years at of 22 assessment centers across the UK between 2006 and 2010.

For details on the donor recruitment and collection of tissue specimens in the GTEx project please see the publication by Carithers et al. (10.1089/bio.2015.0032).

**Ethics oversight**

All FinnGen participants gave written informed study-specific consent. Patients and control subjects in the FinnGen Study provided informed consent for biobank research, based on the Finnish Biobank Act. Alternatively, older Finnish research cohorts, collected prior to the start of the FinnGen Study (August 2017), were collected based on study-specific consents and later transferred to the Finnish biobanks after approval by Fimea, the National Supervisory Authority for Welfare and Health. Recruitment protocols followed the biobank protocols approved by Fimea. The Coordinating Ethics Committee of the Hospital District of Helsinki and Uusimaa (HUS) approved the FinnGen Study protocol No. HUS/990/2017.

The FinnGen project is approved by Finnish Institute for Health and Welfare (THL), approval number THL/2031/6.02.00/2017, amendments THL/1101/5.05.00/2017, THL/341/6.02.00/2018, THL/2222/6.02.00/2018, THL/283/6.02.00/2019), Digital and population data service agency VRK43431/2017-3, VRK/6909/2018-3, the Social Insurance Institution (KELA) KELA 58/522/2017, KELA 131/522/2018, KELA 70/522/2019 and Statistics Finland TK-53-1041-17.
The Biobank Access Decisions for FinnGen Study samples and data utilized in FinnGen Data Freeze 4 include: THL Biobank BB2017_55, BB2017_111, BB2018_19, BB_2018_34, BB_2018_67, BB2018_71, BB2019_7 Finnish Red Cross Blood Service Biobank 7.12.2017, Helsinki Biobank HUS/359/2017, Auria Biobank AB17-5154, Biobank Borealis of Northern Finland_2017_1013, Biobank of Eastern Finland 1186/2018, Finnish Clinical Biobank Tampere MH0004, Central Finland Biobank 1-2017, and Terveystalo Biobank STB 2018001.

UK Biobank obtained ethical approval from the North West Multi-Centre Research Ethics Committee (MREC) (11/NW/0382) to collect and disseminate data and samples from participants and all participants gave informed consent for their genotype data for research use. UK Biobank VV GWAS summary statistics used for analyses carried out in this study came from a study by Ahmed et al. (doi.org/10.1038/s41467-022-30765-y) which was conducted under UK Biobank study no. 22572.

No informed written consent was obtained from GTEx participants as they were deceased when their tissue specimens were

collected. For details on the authorisation of GTEx as a study please see the Consenting Donors section in the Materials and Methods section of Carithers et al. (10.1089/bio.2015.0032)

Note that full information on the approval of the study protocol must also be provided in the manuscript.

# Field-specific reporting

Please select the one below that is the best fit for your research. If you are not sure, read the appropriate sections before making your selection.

☒ Life sciences ☐ Behavioural & social sciences ☐ Ecological, evolutionary & environmental sciences

For a reference copy of the document with all sections, see nature.com/documents/nr-reporting-summary-flat.pdf

# Life sciences study design

All studies must disclose on these points even when the disclosure is negative.

| | |
|---|---|
| Sample size | The study is based on FinnGen Data Freeze 5 (total n = 218,792 individuals) and for the VV analyses, the FinnGen data consisted of 17,027 (13,045 females, 3,982 males) VV cases and 190,028 vein and lymphatic disease-free individuals as controls of VV. The exclusion criteria are described in detail in Methods of the manuscript.<br><br>Ahmed et al. (doi.org/10.1038/s41467-022-30765-y) computed VV variant association summary statistics using 401,656 UK Biobank individuals. |
| Data exclusions | Exclusion of samples and variants was based on standard guidelines and quality control procedures and are described in Methods of the manuscript. The exclusion criteria were pre-established. |
| Replication | Of the total 50 independent genetic loci reported in the present study we included replication tests for 37 of the sex-unspecific lead variants common for both the FinnGen Study and UK Biobank data by Ahmed et al. (https://doi.org/10.1038/s41467-022-30765-y). Out of these 37 lead variants 33 replicated in the UK Biobank data with a P < 1.3e-3 (Bonferroni-corrected P-value threshold corrected for 37 tests) (see Supplementary Data 1). |
| Randomization | The study does not include allocation of participants to different experimental groups. |
| Blinding | No blinding was necessary for this observational study. |

# Reporting for specific materials, systems and methods

We require information from authors about some types of materials, experimental systems and methods used in many studies. Here, indicate whether each material, system or method listed is relevant to your study. If you are not sure if a list item applies to your research, read the appropriate section before selecting a response.

## Materials & experimental systems

| n/a | Involved in the study |
|---|---|
| ☒ | ☐ Antibodies |
| ☒ | ☐ Eukaryotic cell lines |
| ☒ | ☐ Palaeontology and archaeology |
| ☒ | ☐ Animals and other organisms |
| ☒ | ☐ Clinical data |
| ☒ | ☐ Dual use research of concern |

## Methods

| n/a | Involved in the study |
|---|---|
| ☒ | ☐ ChIP-seq |
| ☒ | ☐ Flow cytometry |
| ☒ | ☐ MRI-based neuroimaging |
